# Outcome analysis of primary breast cancer patients who declined adjuvant chemotherapy—results from the prospective multi-center BRENDA II study

**DOI:** 10.1007/s12282-021-01321-1

**Published:** 2022-02-18

**Authors:** Elena Leinert, Lukas Schwentner, Wolfgang Janni, Achim Wöckel, Saskia-L. Herbert, Daniel Herr, Thorsten Kühn, Felix Flock, Ricardo Felberbaum, Rolf Kreienberg, Visnja Fink, Davut Dayan, Kristina Ernst, Susanne Singer, the BRENDA study group

**Affiliations:** 1 Department of Gynaecology and Obstetrics, University of Ulm, Prittwitzstr. 43, 89075 Ulm, Germany; 2 Department of Gynaecology and Obstetrics, University of Würzburg, Würzburg, Germany; 3 Department of Gynaecology and Obstetrics, Hospital Esslingen, Esslingen, Germany; 4 Department of Gynaecology and Obstetrics, Hospital Memmingen, Memmingen, Germany; 5 Department of Gynaecology and Obstetrics, Hospital Kempten, Kempten, Germany; 6 Institute of Medical Biostatistics, Epidemiology and Informatics, University Medical Centre, Johannes Gutenberg University Mainz, Mainz, Germany

**Keywords:** Breast cancer, Guideline adherence, Guideline violation, Adjuvant chemotherapy

## Abstract

**Background:**

This study examined 5-year overall, recurrence and distant metastasis-free survival (OS, RFS, MFS) of high- and intermediate-risk breast cancer (BC) patients who declined guideline-recommended adjuvant chemotherapy (CHT).

**Methods:**

In the prospective multicenter cohort study BRENDA II, patients with primary BC were sampled over a period of four years (2009–2012). A multi-professional team (tumorboard) discussed recommendation for adjuvant CHT according to the German guideline. Potential differences in 5 year survival were analyzed using Kaplan–Meier curves and Cox regression. The hazard ratios (HR) were adjusted for age, Charlson Comorbidity Score, American Society of Anesthesiologist (ASA) physical status classification, and endocrine therapy.

**Results:**

A total of 759 patients were enrolled of which 688 could receive CHT according to the guidelines (*n* = 219 had a clear indication, in *n* = 304 it was possible). For 360 patients, the tumorboard advised to perform CHT, for 304 it advised against and in 24 cases, no decision was documented. Of those with a positive suggestion, 83% received CHT. Until 5 years after diagnosis, 57 patients were deceased, 41 had at least one distant metastasis and 29 a recurrence. There was no evidence for differences in OS and MFS in patients who declined CHT despite tumorboard recommendation (HR 3.5, 95% CI 0.8–15.1 for OS, HR 1.9, 95% 0.6–6.6 for MFS). Patients who received CHT had significantly better 5-year RFS compared to those who declined (HR 0.3, 95% CI 0.1–0.9, *p *= 0.03). There was no evidence for different survival in those who had no CHT because of comorbidity and those who declined actively, neither for OS, MFS nor RFS.

**Conclusion:**

The prospective BRENDA II study demonstrates benefit in RFS by guideline adherence in adjuvant breast cancer treatment, indicating prospectively the value of internationally validated guidelines in breast cancer care.

## Introduction

Women with primary diagnosis of early breast cancer (BC) all in all have a favorable prognosis with good survival rates [1]. Consensus recommendations and guidelines such as the St. Gallen international expert consensus and the German interdisciplinary S3 guideline on diagnosis and treatment of BC were implemented to standardize adjuvant BC therapy and to improve the quality of care [2, 3]. Previous retrospective studies could demonstrate that patients with guideline-adherent adjuvant treatment will have better OS and DFS compared to patients with guideline violations [4–6]. Nevertheless, some patients may decline the recommended standard treatments and are consequently not treated according to guidelines. Regarding CHT, basically two groups can be distinguished among patients who are not treated guideline conform: on the one side, patients with comorbidities or elderly patients who are not accessible for CHT [7] and on the other side, patients that are eligible for CHT but who decline by themselves. However, this second group of patients is the only one for which the decision against CHT could be modified [8, 9]. Therefore, the aim of the prospective BRENDA II study was to assess these patient-related factors that prevent patients from receiving guideline-adherent treatment. Thus, this study aims to answer the following questions:

1. Among BC patients for whom adjuvant CHT is indicated and for whom the tumorboard recommends CHT, do those who do not receive CHT because they decline have worse outcome than those who receive it (after adjusting for comorbidities)?
2. Does the reason for omission of CHT (comorbidities, patient declines herself) make a difference regarding the outcome (in terms of OS, RFS and MFS, adjusted for age)?

## Patients and methods

### Data Collection

In the prospective multicenter cohort study BRENDA II (“Breast Cancer under Evidence-Based Guidelines“), patients with primary BC were sampled consecutively over a period of 4 years (2009–2012). Patients were approached before surgery (t1), before initiation of adjuvant treatment (t2), after completion of adjuvant radio- and/or CHT (t3), and contacted again 5 years after surgery (t4). Patients were eligible for this study if they had been diagnosed with primary histologically confirmed BC. Exclusion criteria were as follows: metastatic or recurrent disease at baseline (including secondary malignancies), bilateral BC, primary occult disease and phylloides tumor, inability to complete a questionnaire and no written informed consent. Each patient was informed about the study by her doctor and asked to participate. If she agreed, the doctor handed over the first series of questionnaires and interviewed the patient. Follow-up interviews were performed by trained study nurses. We collected data at the University Medical Center in Ulm, Kempten Hospital, Memmingen Hospital and Esslingen Hospital, all of which are BC centers certified by the German Cancer Society. Ethical approval was obtained from the Ethics Committee of Ulm University. After surgery, a multi-professional team (tumorboard) consisting of gynecologic, medical, and radiation oncologists, BC surgeons, pathologists, radiologists,and study nurses discussed the recommendation for adjuvant CHT based on current validated guidelines and this decision was documented in a database. Six months later, it was documented whether the patient had received adjuvant CHT or declined.

### Instruments

Clinical data were obtained from the medical records by trained data managers. Co-morbid somatic diseases were assessed by the doctor in charge of the patient’s treatment, documented and subsequently coded according to the Charlson Co-morbidity Index for assessing severe chronic somatic diseases [10]. As further measures of comorbidity, the American Society of Anesthesiologists (ASA) score for physical status was collected for all patients at the time of surgery. Adherence to the tumorboard recommendation was established by comparing the treatment decision, taken by the tumorboard and documented by physicians, with the subsequently received CHT. We used the German national S3 guideline for diagnosis, treatment, and follow-up care in breast cancer (2008 version) to classify the indication for CHT [3]. It has been previously demonstrated by Wolters et al. that adjuvant CHT recommendations do not differ in internationally validated evidence-based guidelines [11]. The classification of risk group was based on the St. Gallen criteria taking into consideration that at the time the national S3 guideline adapted the criteria for the risk groups according to St. Gallen [2, 3].

### Statistical analysis

Absolute and relative frequencies of treatment decisions regarding CHT and subsequent application of CHT were calculated overall and per institution. Survival time was defined as time between baseline (t1) until the event of interest (death, recurrence, distant metastasis) happened or until end of follow-up. If a patient was lost to follow-up, data were censored at the date of the last known contact. Survival distributions and median survival times were estimated using the Kaplan–Meier product-limit method. The 5 year OS rate as well as RFS and MFS with 95% confidence interval (95% CI) were computed using Kaplan–Meier product-limit survival probabilities at the specified time points. Groups were compared using Cox proportional hazards models adjusted for age, Charlson Comorbidity Score, ASA, and endocrine therapy estimating the hazard ratio (HR) and 95% confidence intervals. The proportional hazards assumption was tested using log–log plot and predicted survival plots. Statistical analyses were performed using STATA 12 (StataCorp 2011, College Station, TX, USA: StataCorp LP).

## Results

**Fig. 1:**
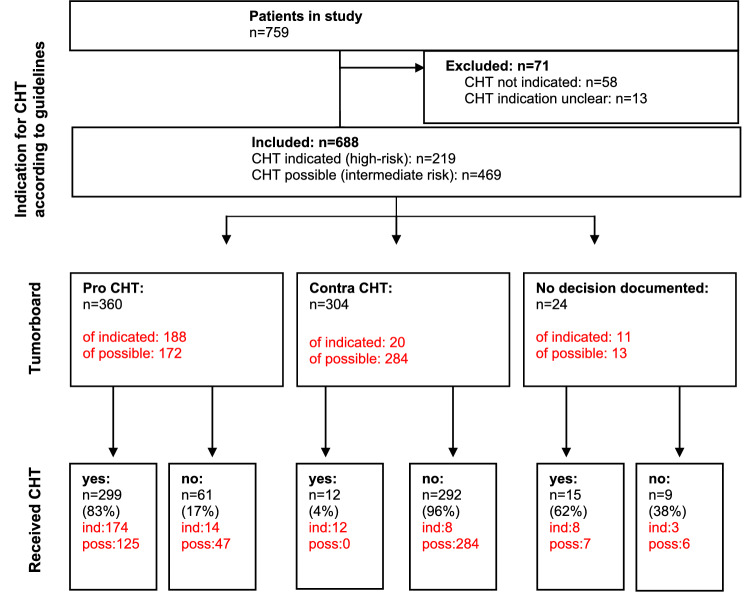
Patient flow through the study

Altogether, 759 patients with primary BC were included in the study. Based on the St. Gallen (2007) criteria and guideline recommendations, 219 patients had clear indication for CHT (high-risk patients), among them 100 patients with Her2-positive BC. A total of 469 patients were intermediate-risk patients, for whom it was possible but not mandatory to recommend adjuvant CHT (Fig. 1). 71 patients were excluded because they had no indication for CHT or indication was unclear. Thus, the entire sample included 688 patients.

We found locally advanced BC (≥ T2, N1) in 65% of the patients (Table 1). 80% of the patients were hormone receptor positive and consequently eligible for endocrine treatment. The tumorboard decision was in favor of CHT in 360 patients (among them 188 high-risk patients and 172 intermediate-risk patients). In 304 of the cases (44%), the tumorboard recommendation was against CHT (20 high-risk patients and 284 at intermediate risk). This proportion differed between centers: the tumorboard decided against CHT in 48%, 37%, 30%, and 33% of the patients (*p *< 0.001).

**Table 1:** Baseline characteristics of the study population (*n* = 688)

	*n*	Percent
Age at diagnosis in years		
< 40	39	6%
40–49	146	21%
50–59	190	28%
60–69	187	27%
70–79	107	16%
80 +	19	3%
Year of diagnosis		
2008	19	3%
2009	139	20%
2010	215	31%
2011	311	45%
2012	3	0.4%
Locally advanced (> = T2, N1)		
No	244	35%
Yes	444	65%
ASA (at baseline)		
I	130	19%
II	403	59%
III	137	20%
IV	2	0.3%
Unknown	16	2%
Charlson comorbidity Index (at baseline)		
0	480	70%
1	97	14%
2	46	7%
3	9	1%
4	10	1%
5	4	1%
6	10	1%
8	3	0.4%
Unknown	29	4%
Hormone receptor		
Negative	139	20%
Positive	549	80%
Her2-status		
Negative	588	85%
Positive	100	15%

Among those patients with a tumorboard recommendation in favor of CHT, 299 patients eventually received CHT (83%) and 61 patients (17%) declined (Fig. 1). There was no evidence that this proportion differed between the centers (19%, 9%, 14%, and 6% respectively declined; *p *= 0.19). The proportion of patients who did not receive CHT despite tumorboard recommendation was higher in intermediate-risk patients than in high-risk patients (27% vs. 7%).

Among the patients without a tumorboard recommendation in favor of CHT, 96% of the patient did not receive CHT and 12 patients still received CHT, all of them belonged to the group of high-risk-patients. Of the 219 high-risk patients, 30 (14%) deceased during the follow-up period. The proportion of lethal events in the intermediate-risk group was 6%.

**Fig. 2:**
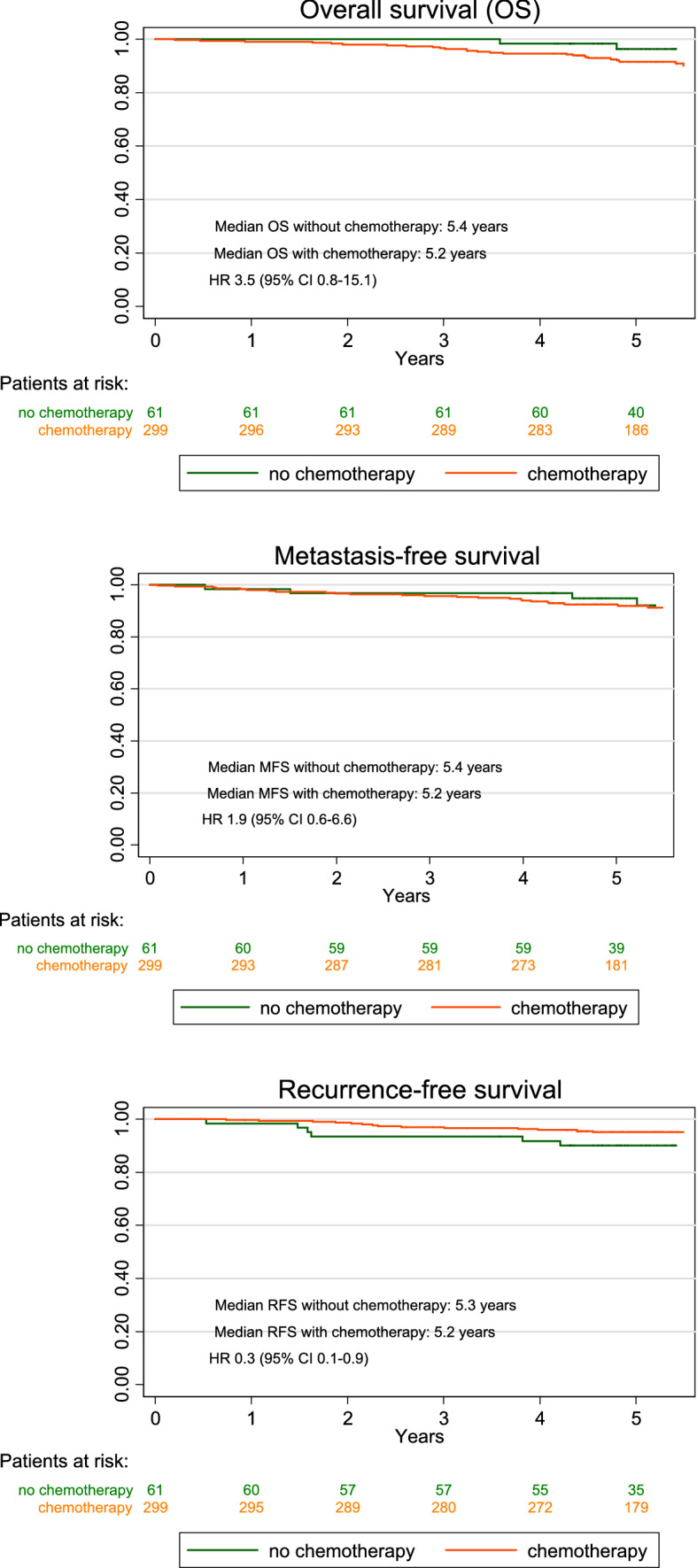
5 year overall survival, metastasis-free survival, and recurrence-free survival in patients with tumorboard recommendation in favor of chemotherapy. Hazard Ratio (HR) adjusted for age, ASA score, Charlson Comorbidity index, endocrine treatment

For further analysis, all patients (high- and intermediate-risk) with a tumorboard recommendation in favor of CHT were combined (*n* = 360). After adjusting for age, ASA score, Charlson Comorbidity index, and endocrine treatment, we found a significant benefit regarding 5 year RFS for patients who received CHT (HR 0.3, 95% CI 0.1–0.9, *p *= 0.03) (Table 2). There was no evidence for differences in OS between patients who received or declined CHT if it was recommended by the tumorboard (HR 3.5, 95% CI 0.8–15.1, *p *= 0.09). The HR for OS was 2.9 in high-risk patients (95% CI 0.4–22.6) and 2.2 in intermediate-risk patients (95% CI 0.2–19.9), but in both groups the confidence intervals were wide and there was no indication of differences in OS for those who received CHT and those who did not. There was also no evidence for differences in MFS (HR 1.9, 95% CI 0.6–6.6, *p *= 0.30) (Fig. 2).

**Table 2:** When the tumorboard had suggested to perform chemotherapy (*n* = 360)

Probability of	Hazard ratio^a^	95% CI	*p* value
Death	3.5	(0.8–15.1)	0.09
In high-risk patients	2.9	(0.4–22.6)	0.30
In intermediate risk patients	2.2	(0.2–19.9)	0.49
Metastasis	1.9	(0.6–6.6)	0.30
Recurrence	0.3	(0.1–0.9)	0.03

Probability of dying, having a distant metastasis, or having a recurrence in patients who received chemotherapy (*n* = 299) versus those who declined chemotherapy (*n* = 61) until 5 years after diagnosis

^a^Adjusted for age, ASA score, Charlson Comorbidity index, endocrine treatment

Another analysis considered patients who did not receive CHT even though it was indicated or possible according to guidelines (*n* = 353). Among them, 22 were high-risk patients. 61 of 353 patients had a recommendation in favor of CHT, but they declined; and in 292 patients, the tumorboard recommended no CHT. Overall, after adjusting for potential confounding factors (age, ASA score, Charlson Comorbidity index, endocrine treatment), there was no evidence for differences in OS, RFS or MFS between those who declined CHT by themselves and those where the tumorboard had advised against CHT. The proportion of deaths was 3% if CHT was recommended by the tumorboard but the patient declined vs. 8% if no CHT had been recommended [HR for OS 0.6 (95% CI 0.1–2.6), *p *= 0.50) (Table 3]. Of the high risk BC patients who did not receive CHT because the tumorboard voted against it, 38% deceased during the follow-up period (Table 4).

**Table 3:** In intermediate and high-risk patients who did not receive chemotherapy (*n* = 353)

Probability of	Hazard ratio^a^	95% CI	*p* value
Death	0.6	(0.1–2.6)	0.50
Metastasis	1.5	(0.4–6.3)	0.56
Recurrence	2.1	(0.6–7.5)	0.25

Probability of dying, having a distant metastasis, or having a recurrence when the patient herself declined chemotherapy (*n* = 61) versus when the tumorboard had recommended not to give chemotherapy (*n* = 292) until 5 years after diagnosis

^a^Adjusted for age, ASA score, Charlson Comorbidity index, endocrine treatment

**Table 4:** Number and proportion of events in the study population (a) overall (b) high-risk patients (c) intermediate-risk patients

(a) Overall							
Tumorboard decision	Pro CHT (*n* = 360)		Contra CHT (*n* = 304)		No decision documented (*n* = 24)		Total
CHT received	Yes	No	Yes	No	Yes	No	
# patients	299	61	12	292	15	9	688
# dead	28 (9%)	2 (3%)	1 (8%)	23 (8%)	1 (7%)	2 (22%)	57
# metastasis	24 (8%)	4 (7%)	2 (17%)	9 (3%)	2 (13%)	0 (0%)	41
# recurrence	14 (5%)	6 (10%)	1 (8%)	7 (2%)	1 (7%)	0 (0%)	29

(b) High-risk patients							
Tumorboard decision	Pro CHT (*n* = 188)		Contra CHT (*n* = 20)		No decision documented (*n* = 11)		Total
CHT received	Yes	No	Yes	No	Yes	No	
# patients	174	14	12	8	8	3	219
# dead	23 (13%)	1 (7%)	1 (8%)	3 (38%)	1 (13%)	1 (33%)	30 (14%)

(c) Intermediate-risk patients							
Tumorboard decision	Pro CHT (*n* = 172)		Contra CHT (*n* = 284)		No decision documented (*n* = 13)		Total
CHT received	Yes	No	Yes	No	Yes	No	
# patients	125	47	0	284	7	6	469
# dead	5 (4%)	1 (2%)	0	20 (7%)	0	1 (17%)	27 (6%)

*CHT* chemotherapy

## Discussion

BC remains a challenge for clinical oncologist, as it represents the most common malignancy in women. Beside operative therapy, radiotherapy and endocrine treatment, CHT is often one essential part of the treatment in early BC setting. The multidisciplinary tumorboard selects the appropriate treatment strategy considering the guidelines on the basis of a risk assessment for each individual patient, in order to avoid both over- and undertreatment regarding CHT. The tumorboard also considers further patient-related factors like age, comorbidities and cognitive impairment. Previous studies could demonstrate that if one or multiple of these factors are constricted, patients will have a higher risk of not receiving guideline-adherent treatment. This finally leads to decreased OS [12–15]. Unfortunately, the reasons why CHT was not recommended by the tumorboard could not be evaluated in this study because of incomplete data. Nevertheless, we suppose that for high-risk patients for whom the tumorboard did not recommend CHT (*n* = 20), the main reasons for omission of CHT were age and/or comorbidities. On the other hand, there is a group of patients (in our cohort 17% both in the high and intermediate risk group), for whom CHT was recommended by the tumorboard but they declined. In the literature, we could find similar rates for both high and intermediate-risk patients who decline CHT [16]. For high-risk patients only, the rate of deviation from CHT was 7% in our cohort, which is also conform to other studies [17]. The proportion of patients who did not receive CHT despite recommendation in favor of CHT was higher in intermediate-risk patients than in high-risk patients. This is most likely due to two reasons: first, we assume that the physician recommends CHT more urgently in high-risk patients and second, patients with intermediate risk BC will have the option to receive endocrine treatment. It is known from other studies that fear of CHT-related side effects is the main reason for omission of CHT from the patient’s point of view [18]. In contrast to age and comorbidities, fear of the treatment would be a modifiable factor to improve treatment adherence. In this regard, more extensive studies would be required to investigate the factors for omission of CHT. In our cohort of high and intermediate risk BC patients, we could find improved RFS after adjuvant CHT but, surprisingly, we could not demonstrate a survival benefit if CHT was applicated. There may be multiple reasons for this result, which is opposite to other studies in early BC setting [19]. One reason may be the very high survival rate in our cohort anyways and with the limited number of cases, a benefit of CHT potentially could not be demonstrated. Particularly in the light of the high proportion of patients with luminal tumors, the follow-up period of 5 years is rather short as relapse often occurs after a longer period [20]. Besides, long-term toxic effects of adjuvant CHT like CHT-induced cardiotoxicity were not considered. Benefits of CHT may be partially obfuscated by adverse effects on the cardiovascular system, resulting in a significant increase in morbidity and mortality [21]. And finally, to our opinion, the main reason is the fact that there were many patients at intermediate-risk in our cohort. For these patients, the rate of recommendation in favor of CHT in our study population was rather high (36%). Nowadays, indication for CHT in primary BC patients depends on intrinsic breast cancer subtype classification as well as clinical parameters such as grading, tumor size or nodal status [22, 23]. However, the data of our study were collected before the era of intrinsic breast cancer subtypes [24]. In a retrospective analysis by Herr et al. on 1376 nodal-positive patients with primary diagnosis of luminal A breast cancer within the BRENDA study, adjuvant CHT in addition to endocrine therapy was not able to improve RFS and tumorspecific OS [25]. Furthermore, Herr et al. demonstrated that tumor size as well as nodal status was not predictive for a benefit of adjuvant CHT in the BRENDA cohort. This is important to note because these parameters were also used for our study population within the framework of the St. Gallen criteria. In the past decade, the use of adjuvant CHT in early BC setting in general decreased [26]. The introduction of gene expression signatures was an important milestone in the treatment intermediate risk BC patients. For that group, studies reported a 20–35% reduction in CHT administration with usage of gene expression signatures [27–29]. On the basis of recent studies, the indication for CHT is increasingly shifting to high-risk BC patients [30]. Thus, one might suspect that if only high-risk patients and patients at intermediate risk with high risk of recurrence had received adjuvant CHT, a survival benefit in the CHT group perhaps could have been demonstrated within the BRENDA study.

In summary, our data provide that patients with high- and intermediate-risk BC have an excellent prognosis and it is the first prospective study demonstrating RFS benefit by guideline adherence in adjuvant breast cancer treatment. This emphasizes the value of internationally validated guidelines in breast cancer care.
